# Integrating Untargeted Metabolomics and Transcriptomics in Mice with Pulmonary Tuberculosis to Reveal Changes in Linoleic Acid and Its Metabolism in Lung Monocyte-Derived Macrophages

**DOI:** 10.3390/pathogens15030254

**Published:** 2026-02-27

**Authors:** Yuxia Sha, Xiaoman Zhao, Hongying Zhu, Ye Li, Meilin Shao, Shenggang Ding, Haoquan Zhou

**Affiliations:** 1 Department of Pediatrics, The First Affiliated Hospital of USTC, Division of Life Sciences and Medicine, University of Science and Technology of China, Hefei 230088, China; yuxiasha@hotmail.com; 2 Anhui Province Key Laboratory of Biomedical Imaging and Intelligent Processing, Institute of Artificial Intelligence, Hefei Comprehensive National Science Center, Hefei 230088, China; zxm1208@iai.ustc.edu.cn (X.Z.); zhuhy62@iai.ustc.edu.cn (H.Z.); 3 Tuberculosis Department 1, Anhui Chest Hospital, Hefei 230022, China; 13966668055@139.com; 4 Department of Physiology, School of Basic Medicine, Anhui Institute of Medicine, Hefei 230601, China; 18009696975@189.cn; 5 Department of Pediatrics, The First Affiliated Hospital of Anhui Medical University, Hefei 230022, China; 6 National Clinical Research Center for Respiratory Disease, Hefei 230022, China

**Keywords:** tuberculosis, monocyte-derived macrophage, transcriptomics, untargeted metabolomics

## Abstract

Pulmonary tuberculosis (TB) remains a major global health challenge. The molecular and metabolic responses of monocyte-derived macrophages (MDMs), which are critical for host defense against *Mycobacterium tuberculosis* (Mtb), are not fully characterized. A murine pulmonary TB model was established by intravenous injection of BALB/c mice with the attenuated Mtb strain H37Ra; controls received saline. After 8 weeks, lung MDMs were isolated for integrated transcriptomic and untargeted metabolomic profiling. Transcriptomic analysis identified 3970 differentially expressed genes (DEGs) in infected MDMs, including upregulated *Ptpn1*, *Dgat2*, and *Alox5ap* and downregulated *Cyld*, *Zfp61*, and *Mapk11*. Metabolomic profiling revealed 113 differentially accumulated metabolites (DAMs). Taurocholic acid and linoleic acid were identified as potential diagnostic biomarkers, both achieving an area under the curve (AUC) of 1.0 in ROC analysis. Integrated omics analysis showed a positive correlation between linoleic acid levels and the expression of *Tbxas1*, *Acaa1b*, and *Acox1*, implicating lipid metabolic pathways in the host response to TB. This multi-omics study delineates key molecular and metabolic alterations in lung MDMs during TB infection. The identified metabolites, taurocholic acid and linoleic acid, show promise as biomarkers, while dysregulated linoleic acid metabolism represents a potential target for novel diagnostic and therapeutic strategies against TB.

## 1. Introduction

Pulmonary tuberculosis (PTB) caused by *Mycobacterium tuberculosis* (Mtb) remains a major global public health challenge. According to World Health Organization (WHO) statistics, this disease accounts for approximately 10 million new confirmed cases and 1.4 million deaths worldwide annually [1]. Mtb primarily infects the lung parenchyma and is transmitted via respiratory droplets, with macrophages acting as the central effector cells in the host immune response against Mtb infection. As a critical physical barrier linking the host to the external environment, the lung harbors a highly heterogeneous macrophage population, whose core subtypes include alveolar macrophages (AMs), interstitial macrophages (IMs), and monocyte-derived macrophages (MDMs). These three subtypes differ significantly in their ontogeny, anatomical localization, phenotypic traits, and functional properties, and consequently exhibit distinctly divergent response profiles to Mtb infection [2,3].

Alveolar macrophages (AMs) originate from hematopoietic progenitor cells in the embryonic yolk sac and fetal liver, colonize the alveolar lumen, and maintain tissue homeostasis through self-renewal, independent of replenishment by peripheral monocytes. They exhibit a phenotypic profile of Siglec-F^+^CD11c^+^CD64^+^MerTK^+^ and serve as the first line of innate immune defense in the alveoli, responsible for clearing pathogens and xenobiotics as well as sustaining alveolar immune homeostasis. Meanwhile, AMs act as the primary host cells for Mycobacterium tuberculosis (Mtb) during early infection: they can exert anti-Mtb activity via reactive oxygen species (ROS) and reactive nitrogen intermediates (RNIs), yet they can also be exploited by Mtb to establish persistent infection [4,5,6]. Interstitial macrophages (IMs) reside in the pulmonary interstitium and possess a dual origin involving both embryonic progenitors and peripheral monocyte-derived replenishment. Characterized by the phenotype of Siglec-F^−^CD11c^+^/^−^CD64^+^CX3CR1^+^, IMs predominantly function to regulate immune homeostasis in the pulmonary interstitium and mediate signal crosstalk between the innate and adaptive immune systems. Upon Mtb infection, IMs secrete proinflammatory cytokines including IL-1β and TNF-α, which recruit immune cells for pulmonary infiltration and initiate local inflammatory responses [7,8]. Monocyte-derived macrophages (MDMs) differentiate from Ly6C^+^CCR2^+^ classical monocytes in the peripheral blood, which are recruited to pulmonary tissues under inflammation-driven conditions. They display the phenotype of CD11b^+^CD11c^+^Siglec-F^−^Ly6C^−^ and are present at extremely low levels in the quiescent state. Following Mtb infection, MDMs undergo rapid population expansion and become the predominant macrophage subset at infectious foci. These cells can exert antibacterial effects and present antigens to activate adaptive immunity; however, their hyperactivation may trigger pulmonary inflammatory necrosis and granuloma formation [3,9]. The functional specialization and heterogeneous responses of distinct pulmonary macrophage subsets constitute the core mechanism governing the balance between anti-infectious immunity and inflammatory injury after Mtb infection. Dysregulation of their functions promotes the progression of tuberculosis from latent infection to active lesions. To date, the metabolic and transcriptional regulatory mechanisms governing MDM function during Mtb infection remain largely unclear.

Omics technologies, including transcriptomics and metabolomics, have become essential tools in tuberculosis research [10]. These approaches enable comprehensive analysis of molecular and cellular changes during infection, facilitating biomarker discovery and mechanistic insights [11,12]. Transcriptomic studies have been used to characterize the immune landscape in tuberculosis [13]. For example, transcriptomic studies have identified distinct activation states and transcriptional profiles in T cells [14], as well as dynamic changes in natural killer (NK) cell function during Mtb infection [15]. Macrophages have also been a focus of omics-based studies, particularly regarding their polarization states and metabolic reprogramming [16,17]. These findings have highlighted shifts from oxidative phosphorylation to glycolysis and the role of lipid metabolism in macrophage responses [18]. However, most research has centered on bulk macrophage populations or alveolar macrophages [3]. The metabolic and transcriptional characteristics of MDMs, despite their central role in bacterial clearance and immune regulation, remain largely uncharacterized in the context of tuberculosis.

Recent studies have begun to shed light on the role of MDMs in tuberculosis. Recruited from circulating monocytes, MDMs acquire distinct phenotypes in the inflamed lung and contribute to bacterial control through production of reactive oxygen and nitrogen species, inflammatory cytokines, and phagolysosomal maturation [19]. They also participate in antigen presentation and modulate adaptive immune responses [20]. Metabolically, MDMs undergo a shift toward glycolysis and exhibit altered lipid metabolism, which supports their immune activity [21]. These findings suggest functional and metabolic distinctions between MDMs and other macrophage subsets. However, most evidence comes from bulk tissue or in vitro studies, and the in vivo metabolic and transcriptional profiles of lung MDMs during tuberculosis remain largely uncharacterized.

To address this gap, a murine model of pulmonary tuberculosis was established using the attenuated H37Ra strain. Transcriptomic and untargeted metabolomic analyses were performed on lung MDMs. By characterizing their molecular and metabolic profiles during infection, this study aims to provide new insights into MDM-specific responses in tuberculosis. Our findings may contribute to a better understanding of disease mechanisms and support the development of improved diagnostic and therapeutic strategies targeting macrophage function.

## 2. Materials and Methods

### 2.1. H37Ra Culture and Identification

H37Ra (Ningbo Mingzhou Biotechnology, Ningbo, China) was streaked onto ATCC Medium 90 7H10 agar plates (M1601; Ningbo Mingzhou Biotechnology, Ningbo, China) and incubated at 37 °C. Acid-fast staining was performed using a G1170 staining kit (Solarbio, Beijing, China) per the manufacturer’s instructions. Under an oil immersion microscope, acid-fast-positive bacteria appeared red, whereas the background and acid-fast-negative bacteria appeared blue.

### 2.2. Establishing a Pulmonary Tuberculosis Mouse Model

Trained professional researchers performed continuous health monitoring of all mice throughout the experiment: general condition assessments were conducted once daily before H37Ra infection, and the frequency was increased to twice daily post-infection. Monitoring parameters included general physiological status, body weight, and Mtb infection-associated pathological symptoms; mice exhibiting abnormal conditions were individually flagged and subjected to an increased frequency of observations. Well-defined criteria for humane endpoints were established based on the pathological characteristics of the Mtb-infected mouse model and national animal ethics guidelines. Mice that met the humane endpoints were immediately euthanized via cervical dislocation, with carcasses disposed of in strict accordance with standardized experimental protocols. The aforementioned animal monitoring protocols and humane endpoint criteria were formally reviewed and approved by the Institutional Animal Care and Use Committee (IACUC) of the First Affiliated Hospital of University of Science and Technology of China (Anhui Provincial Hospital) (Approval No.: 2025-N(A)-042).

Male Balb/c mice aged 6–8 weeks (Vital River Laboratories, Beijing, China) were housed under specific pathogen-free (SPF) conditions with a controlled temperature of 22–25 °C, relative humidity of 40–60%, and a 12-h light/dark cycle. Food and water were provided ad libitum. After five days of acclimation, the mice were randomly divided into two groups (n = 6 per group): the experimental group and the control group.

Tail vein injection is a widely recognized and commonly used method for establishing tuberculosis infection in mouse models [22,23]. In this study, the experimental group received tail vein injections of *Mycobacterium tuberculosis* H37Ra (ATCC 25177), prepared as follows: Colonies cultured on solid medium for 21 days were picked and homogenized in sterile saline containing Tween-80 using grinding rods in 1.5 mL EP tubes. The suspension was quantified using a McGill turbidimetric tube (OD625 = 0.120), corresponding to a concentration of 1.5 × 10^9^ CFU/mL. After gradient dilution, a final inoculum of 2 × 10^6^ CFU in 200 μL was administered per mouse. Mice in the control group received an equal volume of sterile saline via the same route.

After 8 weeks, the mice were anesthetized via intraperitoneal injection of sodium isopentobarbital (60 mg/kg; ≤0.2 mL/10 g body weight). Left ventricular perfusion was then performed using sterile saline until the lung perfusate became clear. Lung tissues were then carefully dissected and fixed in 4% paraformaldehyde for subsequent histological analysis.

### 2.3. Determination of Tissue Colony-Forming Unit (CFU)

To verify the stability of infection load and biological significance of the H37Ra tail vein injection model, an additional 10 6–8-week-old Balb/c mice were selected and randomly assigned to two groups with 5 mice per group (experimental group and control group). All mice were inoculated with H37Ra via the tail vein at a dose of 1 × 10^6^ CFU per mouse, and euthanized at 8 weeks post-infection. Lung tissues were aseptically dissected and homogenized in PBST supplemented with 0.05% Tween 80. After homogenization, the tissue homogenates were serially diluted and plated onto ATCC Medium 90 7H10 agar plates. The plates were incubated at 37 °C for 21 days, and the CFU were finally enumerated for quantitative analysis.

### 2.4. Hematoxylin and Eosin (H&E) Staining

Formalin-fixed, paraffin-embedded lung tissues were sectioned at 5 μm thickness. Sections were dewaxed in xylene, rehydrated through a descending alcohol gradient, and stained with hematoxylin. After differentiation with acid alcohol, nuclei were blued with ammonia water. Cytoplasmic components were stained with eosin, followed by dehydration through graded alcohols and clearing in xylene. Slides were sealed with neutral resin and imaged using a digital slide scanner. Tissue morphology was assessed based on the contrast between blue-purple nuclei and pink cytoplasm.

### 2.5. Preparation of Lung Tissue Single-Cell Suspensions

Lung tissues were rinsed with ice-cold phosphate-buffered saline and mechanically minced with ophthalmic scissors in a chilled Petri dish. The minced tissues were then digested in 2.5 mg/mL collagenase IV and 0.6 mg/mL DNase I at 37 °C with shaking for 45 min. Finally, the mixture was filtered through a 75-μm cell strainer to obtain single-cell suspensions for subsequent antibody staining and purification.

### 2.6. Purification of MDMs

Single-cell suspensions were stained with Fixable Viability Stain 510 (BD Biosciences, San Jose, CA, USA) to exclude dead cells. Cells were incubated with fluorescently labeled antibodies on ice in the dark for 30 min. These antibodies included FVS510 (AmCyan, cat: 564406), CD45 (APC-Cy7, clone: 30-F11), F4/80 (DAPI, clone: BM8), CD11c (PerCP-Cy5.5, clone: HL3), CD11b (PE-Cy7, clone: m1/70), Siglec-F (PE, clone: E50-2440), and Ly6C (BV605, clone: HK1.4rMAb), all obtained from BD Biosciences. Based on the logic of flow cytometry sorting and the specificity of cell subsets, the final marker panel for MDMs was defined in this study as: FVS510^−^CD45^+^F4/80^+^CD11b^+^CD11c^−^/lowSiglec-F^−^Ly6C^+^. MDMs were sorted using a BD FACSAria III flow cytometer and analyzed using FlowJo software (v.10).

### 2.7. Transcriptomic Sequencing of MDMs

Sorted pulmonary MDMs (n = 3 biological replicates per group, with each replicate comprising an independently pooled sample of MDMs isolated from 5 syngeneic mice) were subjected to transcriptomic analysis. Total RNA was extracted using TRIzol reagent (Invitrogen, Carlsbad, CA, USA). RNA quality was evaluated using an Agilent 2100 Bioanalyzer (Agilent Technologies, Santa Clara, CA, USA) and a Nanodrop 2000 spectrophotometer (Thermo Fisher Scientific, Waltham, MA, USA) with RNase-free agarose gel electrophoresis. Next, mRNA was enriched using mRNA capture beads, fragmented, and reverse-transcribed into cDNA. After end repair, poly-A tailing, and adapter ligation, libraries were amplified and sequenced on an Illumina NovaSeq X Plus platform (Guangzhou Gene Denovo Biotechnology, Guangzhou, China).

### 2.8. Transcriptomic Analysis

Raw data were processed using Fastp (v.0.18.0). Adjusted P-values were employed in all subsequent analyses, which were performed using the DESeq2 software package (version 1.38.3). Specifically, the Benjamini–Hochberg (BH) method was applied to conduct multiple test correction, and the adjusted P-values generated therefrom correspond to the controlled false discovery rate (FDR). Differentially expressed genes (DEGs) were identified using DESeq2 with thresholds of *FDR* < 0.05 and |log2FC| > 1. Functional enrichment analyses, including Kyoto Encyclopedia of Genes and Genomes (KEGG) analysis, gene ontology (GO) analysis were conducted to interpret the biological relevance of DEGs. GO analysis covered three functional categories: biological process, cellular component, and molecular function. DEGs were annotated with GO terms using the GO database (http://www.geneontology.org/ (accessed on 18 November 2025)), and enrichment was assessed using a hypergeometric test. The *p*-value was calculated based on the overlap between DEGs and genes annotated to a specific GO term relative to all annotated genes in the background. The resulting *p*-values were adjusted for multiple comparisons using the Bonferroni method, with corrected *p* < 0.05 considered statistically significant. Similarly, KEGG pathway analysis was performed by mapping DEGs to KEGG pathway terms (https://www.genome.jp/kegg/ (accessed on 18 November 2025)) and evaluating enrichment significance using the same statistical framework. Pathways with corrected *p* < 0.05 were regarded as significantly enriched. All analyses were conducted using an established online platform (http://www.omicsmart.com (accessed on 18 November 2025)).

### 2.9. Metabolomic Analysis of MDMs with UHPLC/MS

Sorted pulmonary MDMs (n = 6 biological replicates per group, with each replicate corresponding to an individual MDM sample isolated from a single mouse; no sample pooling was performed to preserve inherent metabolic heterogeneity among individual mice) were subjected to metabolomic profiling. Cells were lysed in an extraction buffer. After multiple freeze–thaw cycles and centrifugation (12,000 rpm, 15 min, 4 °C), supernatants were analyzed using an Elute UHPLC system (Bruker Daltonics, Bremen, Germany) with an ACQUITY UPLC BEH C18 column (100 × 2.1 mm, 2.0 μm). Chromatographic separation was performed using 0.1% formic acid in water (mobile phase A) and acetonitrile (phase B) under a gradient elution: 1–99% B over 13 min, with a flow rate of 0.3 mL/min, column temperature at 40 °C, and injection volume of 3 μL. Metabolites were detected in both ion modes using a timsTOF Pro 2 mass spectrometer (Bruker Daltonics, Bremen, Germany) with the following parameters: 70–1300 Da range, capillary voltages +4500 V/−3600 V, nebulizer pressure 2.2 bar, dry gas 10.0 L/min, temperature 220 °C, and auto MS/MS with 0.32 s cycle time. Quality control (QC) samples were prepared by pooling equal aliquots from all samples. Mass and mobility calibration was achieved using the internal calibration solution tune mix and Na formate (*v*/*v*, 7:3).

### 2.10. Metabolomic Analysis

Data were processed using SIMCA v.16.0.2 software (Sartorius Stedim Biotech, Göttingen, Germany). were conducted. The coefficient of variation (CV) of all quality control (QC) samples was <30%. The raw data was processed using Bruker MetaboScape 2022 (version 2022a) software for quality calibration, peak extraction, alignment, filtering, and annotation. Substance annotation was performed through database matching using the Bruker HMDB Metabolite Library 2.0, Bruker MetaboBASE Personal Library 2.0 and 3.0, and the NIST 2020 Metabolite Library for secondary mass spectrometry. The annotation parameters were set as follows: *m/z* < 2 ppm, mSigma < 20, MS/MS score > 850 (highly confident), *m/z* < 10 ppm, mSigma < 500, and MS/MS score > 400 (confident). Data were normalized using total ion current and imported into SIMCA v16.0.2 for statistical analysis. After log transformation and Pareto scaling, principal component analysis (PCA) was performed to visualize sample distribution and detect outliers within a 95% confidence interval. To distinguish metabolic profiles between groups, orthogonal partial least squares discriminant analysis (OPLS-DA) was applied. Model reliability was evaluated using 7-fold cross-validation and 200 permutation tests. Differentially accumulated metabolites (DAMs) were defined by VIP > 1 and *p* < 0.05. Identified DAMs were subjected to KEGG pathway enrichment using MetaboAnalyst 5.0. ROC curve analysis was used to assess potential biomarkers, and Pearson correlation analysis was performed to evaluate associations between metabolites and inflammatory cytokines.

### 2.11. Quantitative Real-Time Polymerase Chain Reaction

1 μg of total RNA was used for cDNA synthesis (reverse transcription kit: Evo M-MLV RT Premix kit, AG11706-S; Accurate Biotechnology, Changsha, Hunan, China), so as to ensure a sufficient amount of template and a uniform concentration for the reverse transcription reaction. After cDNA was synthesized, quantitative real-time polymerase chain reaction (qRT-PCR) was performed with SYBR Green Premix Pro Taq HS (AG11701-S; Accurate Biotechnology) on a LightCycler 480 Instrument II (Roche Diagnostics) under the following cycling conditions: 95 °C for 5 s followed by 60 °C for 30 s (40 cycles). This study comprised two experimental groups, with five biological replicates assigned to each group (corresponding to five pooled samples, where each pooled sample contained 5 mice from the same batch). Three technical replicates were performed for each biological replicate to eliminate systematic experimental errors. All primers were designed and synthesized by General Biology Co., Ltd. (Chuzhou, Anhui, China). Table 1 presents the primer sequences employed in the qRT-PCR assays, and melting curve analysis was subsequently conducted immediately after the completion of qRT-PCR reactions. The levels of mRNA were normalized to GAPDH by using the 2^−ΔΔCT^ method.

### 2.12. Statistical Analysis

Data were analyzed using GraphPad Prism v.10 software (GraphPad Software Inc. San Diego, CA, USA). The Shapiro–Wilk test was used to verify data normality. The results are presented as mean ± standard error of the mean. All *p*-values were determined via two-tailed tests. A *p* value of less than 0.05 was considered statistically significant. In this study, the Pearson correlation test was used for gene-metabolite correlation analysis, and the Benjamini–Hochberg (BH) method was applied to adjust P-values for multiple testing to control the *FDR*. An *FDR* value of less than 0.05 was considered statistically significant.

## 3. Results

### 3.1. Establishing a Pulmonary Tuberculosis Mouse Model and Isolating Lung MDMs

**Figure 1:**
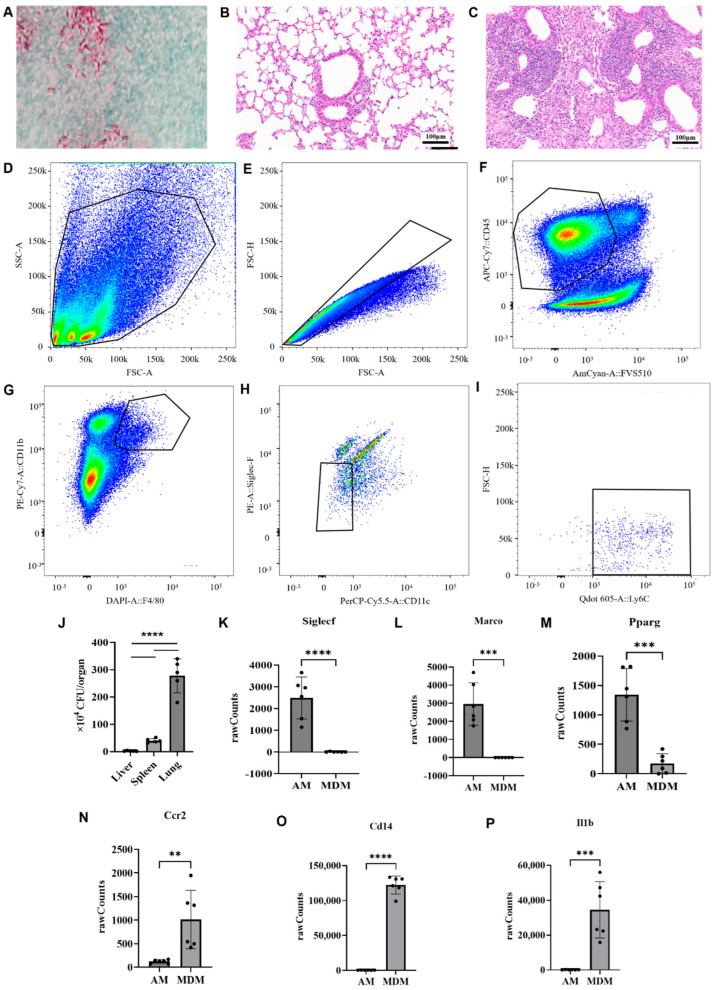
Validation of our pulmonary tuberculosis mouse model. (**A**) Acid-fast staining results of H37Ra for bacterial identification. (**B**) H&E-stained lung tissue section from a healthy control mouse. (**C**) H&E-stained lung tissue section from a tuberculosis mouse model established through H37Ra tail vein injection. (**D**–**I**) Flow cytometry gating strategy for isolating lung monocyte-derived macrophages (MDMs). Group labels (left) show control (top panels) and experimental (bottom panels). Plots (left to right) display all cells, singlets after de-adhesion, CD45^+^ live cells, and gated MDMs. (**J**) At 8 weeks post-infection, the bacterial load in the liver was (2.26 ± 1.40) × 10^4^ CFU per organ, and that in the spleen was (4.06 ± 0.78) × 10^5^ CFU per organ; the bacterial load in lung tissues reached (2.78 ± 0.63) × 10^6^ CFU per organ (**J**). These findings demonstrated a markedly lower bacterial burden in extrapulmonary organs (liver and spleen) relative to the lung, thus confirming that this established model is a lung-predominant Mtb infection model. n = 5. (**K**–**P**) Comparison of the expression profiles of signature markers between MDMs and alveolar macrophages (AMs) revealed that, relative to AMs, the gene expression levels of AM-specific signature markers (*Siglec*-*F*, *Marco*, *Pparg*) were significantly downregulated in MDMs, whereas those of MDM signature markers (*Ccr2*, *Cd14*, *Il1b*) were markedly enriched. Data are presented as the mean ± SEM. Significance: ** *p* < 0.01, *** *p* < 0.001, and **** *p* < 0.0001 vs. alveolar macrophages (AM).

To investigate the molecular and metabolic responses of MDMs during tuberculosis, a murine model of pulmonary TB was established. Acid-fast staining was performed to verify that H37Ra exhibited the staining characteristic of Mtb (Figure 1A). H&E staining was used to assess pathological changes in the lungs of mice with tuberculosis, revealing epithelioid cell aggregation, early granuloma formation, lymphoid hyperplasia, and incipient tuberculous nodule development (Figure 1B,C). At 8 weeks post-infection, the bacterial load (CFU) in the liver, spleen, and lungs was (2.26 ± 1.40) × 10^4^, (4.06 ± 0.78) × 10^5^, and (2.78 ± 0.63) × 10^6^ per organ, respectively (Figure 1J). This confirms a lung-predominant Mtb infection, as extrapulmonary bacterial burden was markedly lower. Combined with evidence from previous studies [23], the defined dosage of H37Ra administered via tail vein injection in this study results in a stable pulmonary CFU load at 8 weeks post-infection. In addition, hematoxylin and eosin (H&E) staining revealed no tissue damage characteristic of acute severe infection (e.g., extensive necrosis) in lung tissues (Figure 1C). Collectively, these observations confirm that the tuberculosis model established in the present study exhibits a chronic-like disease state. Subsequently, transcriptome sequencing was performed on the sorted MDMs, and the obtained sequencing data were compared with the alveolar macrophage (AM) transcriptome sequencing datasets retrieved from the Gene Expression Omnibus (GEO) database (Accession No.: GSE125287)—with a specific focus on the expression profiles of signature markers for MDMs and AMs (Figure 1K–P). The results demonstrated that, compared with AMs, the gene expression levels of AM-specific signature markers (*Siglec-F*, *Marco*, *Pparg*) were significantly downregulated in MDMs, whereas those of MDM signature markers (*Ccr2*, *Cd14*, *Il1b*) were markedly enriched. These findings confirmed at the transcriptomic level that the sorted cells were bona fide MDMs, and effectively ruled out the major possibility of cross-contamination by alveolar macrophages. These results collectively validated the successful establishment of the pulmonary tuberculosis model and confirmed effective enrichment of lung MDMs for downstream transcriptomic and metabolomic analyses.

### 3.2. QC of Transcriptomic Data

**Figure 2:**
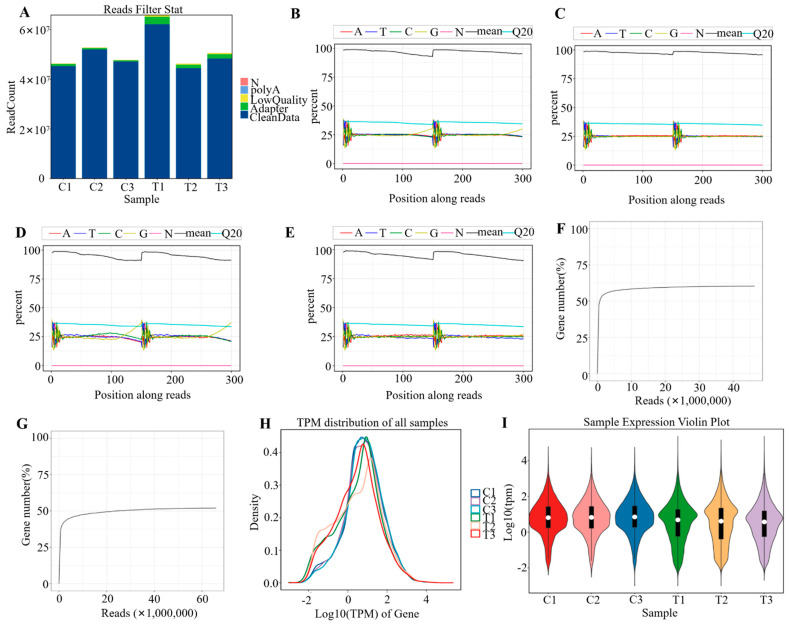
Data filtering statistics. (**A**) Data preprocessing distribution (numerical metrics). (**B**–**E**) Nucleotide composition profiles of samples C1 and T1 before and after quality filtering. (**F**,**G**) Sequencing saturation levels for samples C1 and T1. (**H**) Gene expression abundance distribution across samples. (**I**) Violin plots illustrating gene expression levels (TPM values). This study included two experimental groups, with three biological replicates per group (corresponding to three pooled samples, each containing 5 mice from the same batch; n = 3). The letter C denotes the control group, and T denotes the *H37Ra*-infected group.

With lung MDMs successfully isolated, high-throughput transcriptomic sequencing was performed to investigate gene expression changes during tuberculosis infection. Low-quality sequencing reads were filtered to remove sequences with low-quality bases, adapter contamination, or sequencing errors. Postfiltering statistics revealed that more than 95% of the reads met our quality thresholds (Figure 2A). After low-quality bases were trimmed or removed, base composition and quality score distributions were analyzed. The majority of the base quality scores were found to stabilize at approximately 25, with tight and reliable distributions (Figure 2B–E). Next, sequencing saturation, a key quality metric reflecting the proportion of unique transcripts detected at a given sequencing depth, was evaluated. Because MDMs are typically low-complexity samples, the sequencing saturation levels exceeded 50% across all six samples, indicating sufficient sequencing depth (Figure 2F,G). Genes with transcripts per million (TPM) values below 10 are considered to be of low abundance. Therefore, to evaluate the distributions of transcript abundance, expression density plots (Figure 2H) and violin plots (Figure 2I) were generated. The results indicated that all six samples exhibited concentrated gene expression profiles with minimal variability. In addition, the supplementary data, including the alignment rate (Table S1), percentage of uniquely mapped reads (Table S1), ribosomal RNA (rRNA) alignment rate (Table S2), base quality (Table S3), and saturation analysis (Figure S1), collectively validated the high reliability of the RNA-seq data, and thus laid a solid foundation for subsequent gene expression quantification and correlation analysis. Notably, the violin plots of the experimental group exhibited narrower widths at TPM > 10 compared with those of the control group, suggesting a higher proportion of low-abundance genes in the tuberculosis-infected group. This differential expression pattern may reflect transcriptional changes associated with the pathogenesis of tuberculosis, thereby warranting further biological validation and analysis.

### 3.3. Transcriptome Sample Relationship Analysis and Overall Analysis of Gene Differences Between Groups

**Figure 3:**
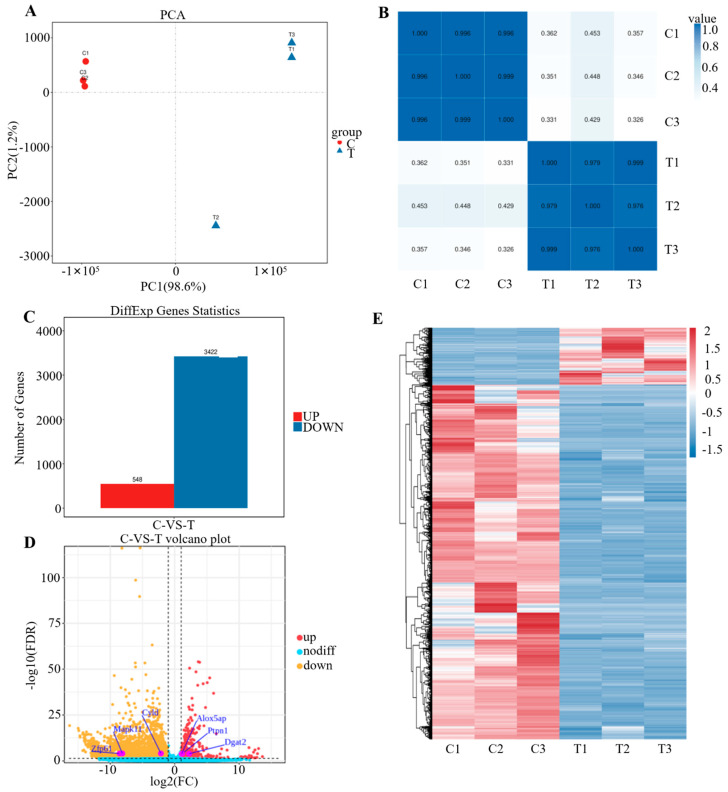
Sample relationship analysis results. (**A**) Sample Principal Component Analysis (PCA) results. (**B**) Sample correlation heatmap. (**C**) Statistical chart of DEGs. (**D**) Volcano plot comparing Differentially Expressed Genes (DEGs). The horizontal axis (*x*-axis) represents the logarithmic fold of gene expression changes (log_2_ fold change). The farther a point is from the center of the graph, the greater the fold difference is in gene expression between the two groups. The vertical axis (*y*-axis) represents the negative logarithm of statistical significance (−log_10_ *P* or −log_10_ adjusted *P*). Points closer to the top of the graph indicate more significant gene expression. Genes with significant upregulation are depicted in red, genes with significant downregulation are depicted in blue, and genes with no significant differences are depicted in gray. (**E**) Heatmap of differential gene clustering. Each color (i.e., red indicating high expression and blue indicating low expression) indicates a unique level of gene expression. Color depth is used to rapidly identify genes with significantly different expression levels in each sample. The letter C denotes the control group, and T denotes the *H37Ra*-infected group.

PCA analysis showed that PC1 and PC2 together explained 99.8% of the total variance. Clear separation between control and experimental groups was observed along PC1, with tight clustering within each group, indicating consistent intra-group gene expression (Figure 3A). Pearson correlation analysis further confirmed strong within-group similarity, with correlation coefficients near 1 among replicates, supporting data reliability and experimental reproducibility (Figure 3B). Differential expression analysis identified 3970 DEGs, including 548 upregulated and 3422 downregulated genes in the experimental group (Figure 3C). Volcano and cluster heatmaps visualized distinct gene expression patterns, providing a basis for further functional studies (Figure 3D,E).

### 3.4. Transcriptome Data Enrichment Analysis

**Figure 4:**
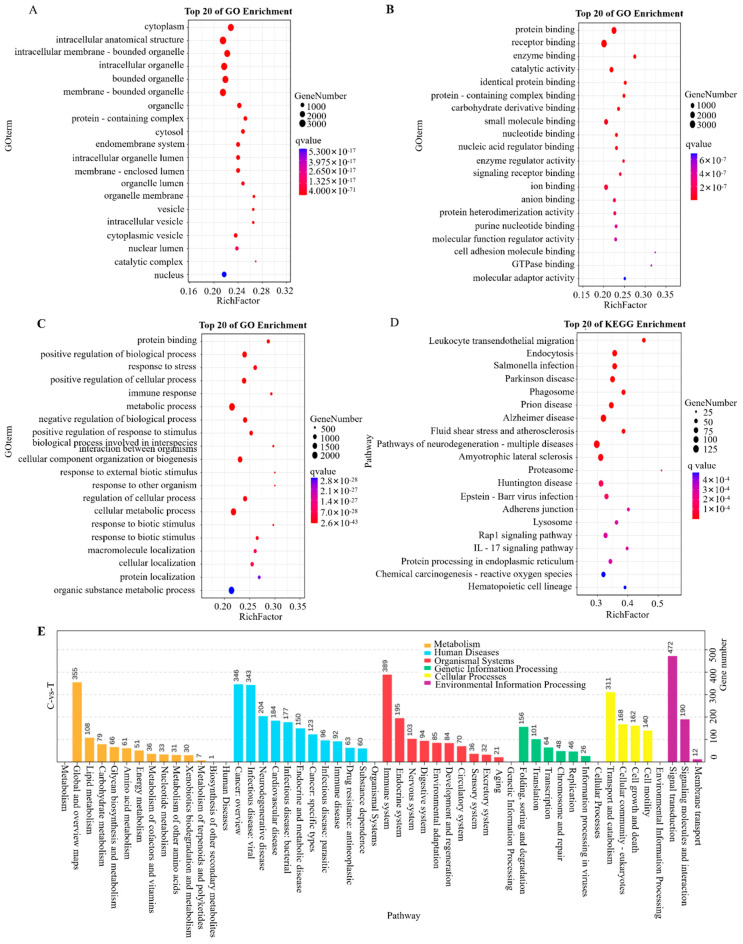
GO enrichment analysis results of transcriptome data. (**A**) Bubble plot of GO-enriched cell components. Larger bubbles indicate more GO-enriched cell components, and redder bubbles indicate smaller *q* values. (**B**) Functional bubble diagram of GO-enriched molecules. (**C**) Bubble plot of GO-enriched biological processes. (**D**) Bubble plot of KEGG enrichment. (**E**) Statistical chart of the number of enriched KEGG genes, where the horizontal axis represents the number of enriched KEGG genes in each pathway and the vertical axis represents KEGG hierarchical classification. The letter C denotes the control group, and T denotes the *H37Ra*-infected group.

To further explore the biological relevance of DEGs, GO enrichment analysis was performed across three categories: biological processes, molecular functions, and cellular components. The top 20 enriched terms revealed significant involvement in immune regulation, metabolic processes, response to stimuli, and cellular localization (Figure 4A–C). To complement these findings, KEGG pathway analysis was conducted, showing that DEGs were enriched in pathways related to metabolism (particularly lipid metabolism), immune function, and signal transduction, including Rap1 signaling pathway and IL-17 signaling pathway (Figure 4D,E).

### 3.5. qRT-PCR Validation of RNA Sequencing Data

**Figure 5:**
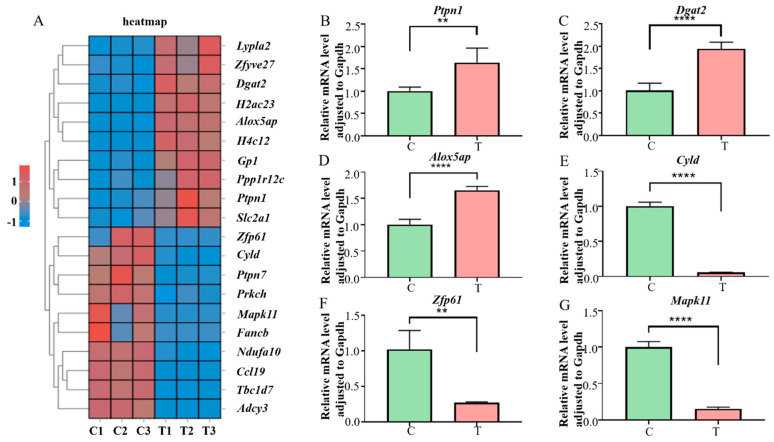
Validation results of data generated by RNA sequencing through qRT-PCR. (**A**) Heatmap of the top 10 significantly upregulated DEGs and top 10 significantly downregulated DEGs. (**B**–**G**) Expression levels of *Ptpn1*, *Dgat2*, *Alox5ap*, *Cyld*, *Zfp61*, and *Mapk11* determined by qRT-PCR. This study comprised two experimental groups, with five biological replicates per group (corresponding to five pooled samples, each containing 5 mice from the same batch; n = 5). Three technical replicates were performed for each biological replicate to eliminate systematic experimental errors. Data are presented as the mean ± SEM. Significance: ** *p* < 0.01, and **** *p* < 0.0001 vs. the control group. The letter C denotes the control group, and T denotes the *H37Ra*-infected group.

To verify the accuracy of the transcriptome data, the top 10 significantly upregulated DEGs and top 10 significantly downregulated DEGs (Figure 5A) were analyzed. The Shapiro–Wilk test was employed to assess the normality of all experimental data, and the results demonstrated that all data conformed to a normal distribution (*p* > 0.05), thus satisfying the fundamental assumptions for the t-test (Figure S2). Following the qRT-PCR reactions, melting curve analysis was performed, which revealed that all target genes exhibited a single sharp melting peak with no non-specific peaks or primer-dimer signals detected. This confirmed that the amplified products were single, specific amplicons (Figure S3). Among the upregulated genes, *Ptpn1* is involved in intracellular signaling and immune response [24], *Dgat2* is involved in immune response regulation [25], and *Alox5ap* influenced the function of macrophages [26]. Among the downregulated genes, *Cyld* (cylindromatosis) is involved in the regulation of cell signaling and inflammatory response [27], *Zfp61* functions as a transcription factor in gene expression regulation [28], and *Mapk11* (mitogen-activated protein kinase 11), also known as p38-β, is involved in cellular stress response, inflammatory response, and immune response [29]. Overall, these six genes were significantly related to macrophage immunity and had a significant effect on the process of tuberculosis. Therefore, they were analyzed using qPCR to measure their mRNA expression levels. The results revealed gene expression levels consistent with those of RNA sequencing, indicating the accuracy of the RNA sequencing results (Figure 5B–G).

### 3.6. PCA and OPLS-DA of Metabolomic Data

**Figure 6:**
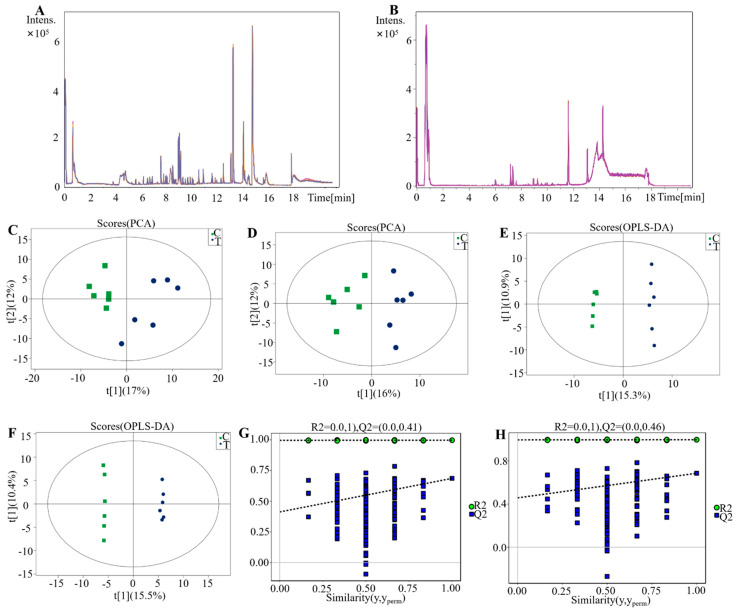
PCA and Orthogonal Projections to Latent Structures-Discriminant Analysis (OPLS-DA) results of metabolomic data. (**A**) BPC overlap plot for UHPLC/MS detection of the QC sample in positive ion mode. (**B**) BPC overlap plot for UHPLC/MS detection of the QC sample in negative ion mode. (**C**) Negative PCA results. (**D**) Positive PCA results. (**E**) Negative OPLS-DA results. (**F**) Positive OPLS-DA results. (**G**,**H**) For the permutation test plot of the OPLS-DA model, the *x*-axis denotes the permutation retention rate (namely the proportion of consistent order with the Y variable of the original model), and the *y*-axis represents the values of R^2^ and Q^2^. This study included two experimental groups, with six biological replicates per group; each biological replicate corresponded to an individual mouse from the same batch, i.e., n = 6. Here, C denotes the control group, and T denotes the H37Ra-infected group.

Subsequently, the UHPLC/MS analysis was conducted on MDMs extracted from the lungs of the experimental and control groups. Metabolites were detected in both positive and negative ion modes. The retention time and peak intensity in the BPC overlap plots of the same QC sample batches were found to be consistent, indicating stable instrument performance during the detection process (Figure 6A,B). PCA was performed and revealed a significant metabolic distinction between the two groups (Figure 6C,D). Based on these results, an OPLS-DA model was constructed to further analyze the differences. Combined with the permutation test plot of the OPLS-DA model, both R^2^ and Q^2^ values of the random models decreased progressively as the permutation retention rate declined gradually (Figure 6G,H). This indicated that the original model had no overfitting and exhibited favorable robustness.

### 3.7. DAMs in the Lung MDMs and Screening for Potential Pulmonary Tuberculosis Biomarkers

**Figure 7:**
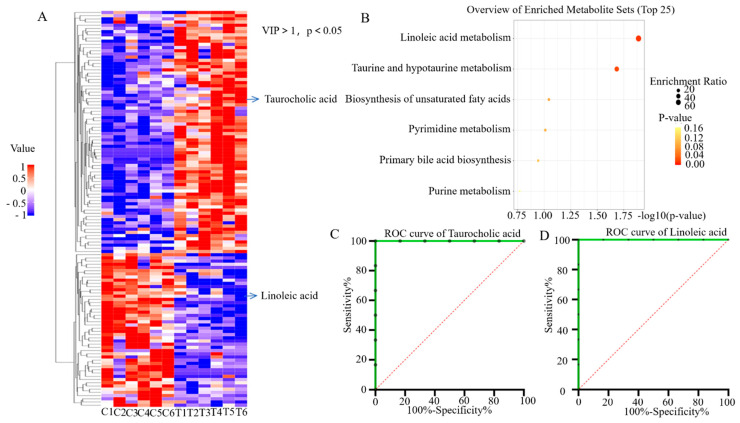
ROC curves of Differentially Abundant Metabolites (DAMs) and pulmonary tuberculosis biomarkers in the lung MDMs of two groups of mice. (**A**) Clustering heatmap. (**B**) Bubble chart. (**C**,**D**) ROC curves of taurocholic acid and linoleic acid as pulmonary tuberculosis candidate biomarkers. It should be noted that the biological matrix used for the detection of the two candidate metabolites (taurocholic acid and linoleic acid) in this study was sorted MDMs. Here, C denotes the control group and T denotes the H37Ra-infected group.

DAMs in both positive and negative ion modes were defined using criteria of *p* < 0.05 and VIP > 1. Clustering heatmap analysis identified 113 DAMs, including 65 upregulated and 48 downregulated metabolites (Figure 7A). These DAMs were categorized into six groups. The first group comprised fatty acids, including saturated and unsaturated fatty acids, such as FA 32:9, FA 26:5, linoleic acid, and heptadecanoic acid, and fatty acid derivatives, such as P-16-0/18:1 (9Z) and 1-oleoyl-2-hydroxy-*sn*-glycero-3-phosphate-(1′-rac-glycerol). The second group comprised amino acids and peptides, including Leu, Arg, Gly, His, and Phe. The third group comprised phospholipids, including phosphatidylserines such as P-16-0/20:4 (5Z, 8Z, 11Z, 14Z) and 16:0/18:3 (9Z, 12Z, 15Z), phosphatidylethanolamines such as 13:1_16:3 and 19:1(9Z)/0:0. The fourth group comprised ceramides, including normal ceramides such as Cer 29:2 (3O/38:2) and Cer 31:3 (2O/32:9) and hexacarbon sugar ceramides such as HexCer 37:3 (2O/32:6) and HexCer 38:0 (3O/26:2). The fifth group comprised heterocyclic compounds, including nitrogen-containing heterocyclic compounds such as 4-(6-methyl-2-pyrolidin-1-ylpyridin-4-yl)-*N*-(3,4,5-trimethoxyphenyl)piperazine-1-carboxamide and [1,3]oxazolo[4,5-*c*]pyridine-2-thiol, and halogenated compounds such as 4,4,4-trifluorobutyric acid and 3-iodo-2-propynylbutylcarbamate. The sixth group comprised other chemicals, including bile acids such as taurocholic acid and other small molecules such as hexaethylene glycol. These metabolites demonstrated various associations with monocytes and tuberculosis. Notably, taurocholic acid was significantly upregulated in the experimental group, whereas linoleic acid was significantly downregulated in the experimental group. KEGG enrichment analysis revealed that significant involvement of DAMs in linoleic acid metabolism and taurocholic acid metabolism (Figure 7B). To assess the biomarker potential of the DAMs, ROC analyses were performed. Both taurocholic acid (Figure 7C) and linoleic acid (Figure 7D) achieved an AUC of 1.0, indicating that these two metabolites act as promising candidate metabolic biomarkers for tuberculosis (TB). It should be noted that the biological matrix used for the detection of the two candidate metabolites (taurocholic acid and linoleic acid) in this study was sorted MDMs. Additionally, these findings were derived from a limited mouse cohort (n = 6 per group) and a single tail vein injection model with the attenuated H37Ra strain; thus, potential issues of overfitting and overestimation of efficacy may exist, and the observed performance cannot be directly extrapolated to the diagnostic efficacy in clinical settings.

### 3.8. Integration Analysis of DEGs and DAMs

**Figure 8:**
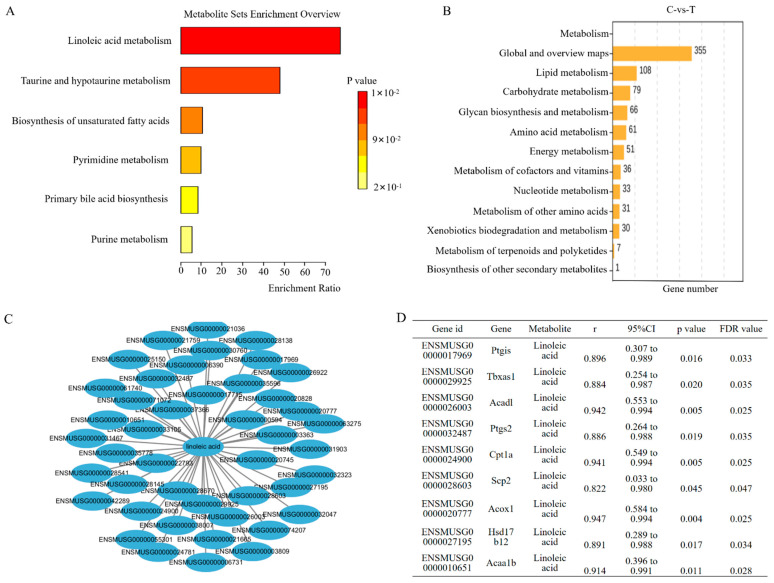
Integrated analysis results of DEGs and DAMs. (**A**) KEGG enrichment bar chart of DAMs. (**B**) KEGG enrichment metabolic pathway entries of DAMs. (**C**) Network graph of genes related to linoleic acid metabolism in DAMs. (**D**) Correlation analysis of genes and metabolites. Here, C denotes the control group and T denotes the H37Ra-infected group.

KEGG pathway enrichment analysis was performed to explore the relationship between DAMs and DEGs. KEGG enrichment showed that DAMs were primarily associated with linoleic acid metabolism, while DEGs were mainly enriched in lipid metabolism pathways, with overlap in linoleic acid metabolism between the two datasets (Figure 8A,B). In this study, two criteria—KEGG pathway annotation and GO functional keywords—were employed to screen for lipid metabolism-related genes. The detailed workflow was as follows: first, all genes annotated to the lipid metabolism pathway were retrieved from the KEGG database. Then, relevant genes were identified from the database using the GO functional keywords: lipid metabolism, fatty acid metabolism, lipid biosynthesis, and linoleic acid metabolism. These two sets of candidate genes were subsequently merged with duplicate entries removed, followed by further screening for lipid metabolism-related genes among the transcriptome-wide differentially expressed genes (FDR < 0.05). Ultimately, 119 lipid metabolism-related genes were identified for subsequent integrative analysis. Two key metabolites related to linoleic acid metabolism were identified, including linoleic acid itself, which has known links to tuberculosis. After Pearson correlation analysis and correction with the Benjamini–Hochberg method, 44 significantly correlated gene-metabolite pairs were identified in this study (Figure 8C and Table S4). Subsequently, based on KEGG pathway annotation and NCBI official pathway association information, nine genes associated with linoleic acid metabolism were curated, among which three genes of particular interest were *Tbxas1* (r = 0.884, FDR = 0.035), *Acaa1b* (r = 0.914, FDR = 0.028), and *Acox1* (r = 0.947, FDR = 0.025) (Figure 8D). However, the conclusions of this study are solely derived from correlation analysis between transcriptomics and metabolomics, and further experimental validation is required to confirm the causal relationships underlying these associations.

## 4. Discussion

Pulmonary tuberculosis is characterized by complex host–pathogen interactions, with monocyte-derived macrophages (MDMs) serving as core target cells for Mycobacterium tuberculosis (Mtb) infection and key mediators of immunometabolic regulation [30,31]. This study performed integrated transcriptomic and metabolomic analyses of pulmonary MDMs in a murine TB model, identifying 3970 differentially expressed genes (DEGs) and 113 differential abundant metabolites (DAMs). Transcriptomic results showed significant upregulation of *Ptpn1*, *Dgat2*, and *Alox5ap*, and downregulation of *Cyld*, *Zfp61*, and *Mapk11*—all involved in immune signaling, inflammation, or lipid metabolism—with qPCR validating these changes. Metabolomic analysis identified taurocholic acid and linoleic acid as candidate biomarkers (AUC = 1.0), and integrated analysis revealed a significant correlation between linoleic acid levels and the expression of lipid metabolism-related genes *Tbxas1*, *Acaa1b*, and *Acox1*. Notably, the high proportion of downregulated DEGs may relate to Mtb’s immune suppressive strategies, as Mtb secretes effector molecules to repress antibacterial and immune activation gene transcription [32], and MDM differentiation in inflammatory microenvironments may downregulate basic metabolic genes to prioritize antibacterial immunity [30]. These findings align with the well-established role of lipid metabolic reprogramming in TB pathogenesis [33].

Among the DEGs, three genes—*Ptpn1*, *Dgat2*, and *Alox5ap*—were significantly upregulated, while *Cyld*, *Zfp61*, and *Mapk11* were significantly downregulated in the experimental group. Ptpn1 encodes a nonreceptor type protein tyrosine phosphatase that regulates intracellular signaling and is involved in inflammation, metabolism, and cell proliferation [34]. PTPN1 expression has been shown to modulate macrophage-mediated inflammation and contribute to organ damage in sepsis [35]. Dgat2 encodes a membrane-bound enzyme essential for triglyceride synthesis, with known proinflammatory effects in macrophages upon LPS stimulation [36]. Alox5ap activates the 5-lipoxygenase pathway, promoting leukotriene biosynthesis and contributing to inflammatory responses [37]. In contrast, among the down-regulated genes, CYLD negatively regulates inflammatory responses by modulating NF-κB, JNK, and Wnt pathways [38]. Mapk11 is part of the p38 MAPK family and controls cytokine production during stress and infection [29]. Zfp61, a zinc finger transcription factor, plays a role in gene expression and immune regulation [39], although its direct link to tuberculosis remains unclear. Collectively, these genes are involved in key signaling pathways, including tyrosine dephosphorylation, lipid synthesis, cytokine production, and ubiquitination, that may contribute to macrophage activation and the progression of tuberculosis [40].

To our knowledge, this is the first integrated transcriptomic and metabolomic analysis of pulmonary MDMs during in vivo Mtb infection, focusing on linoleic acid metabolic alterations. Our findings exhibit both consistency with and distinct novelty and specificity compared to existing metabolomic investigations into pulmonary tuberculosis. This consistency is reflected in the well-established evidence that lipid metabolic reprogramming constitutes a core pathological feature of pulmonary tuberculosis, and that fatty acid metabolic dysregulation is closely linked to the functional regulation of immune cells and the clinical outcomes of Mtb infection [41]. For instance, previous study demonstrated that the levels of unsaturated fatty acids in the peripheral blood of patients with pulmonary tuberculosis are significantly aberrant, and this alteration correlates with inflammatory cytokine expression [42]—an observation that aligns well with our findings regarding the dysregulation of linoleic acid (a core member of ω-6 polyunsaturated fatty acids) and its association with immune regulation. In contrast, the novelty and specificity of our study stand in clear distinction from prior research that has focused on mixed samples including whole blood, whole lung tissue, or bronchoalveolar lavage fluid (BALF) [20,43]. Such mixed samples contain multiple cell types and thus tend to mask the cell-type-specific metabolic characteristics of targeted cells. By precisely focusing on the homogeneous pulmonary MDM cell subset, our study not only identified a specific downregulation pattern of linoleic acid in these cells but also, for the first time, verified a significant positive correlation between linoleic acid levels and the expression of genes including Tbxas1, Acaa1b, and Acox1 in Mtb-infected pulmonary MDMs. This work overcomes the limitations of cellular heterogeneity inherent to traditional studies, thereby providing high-resolution, target cell-level data for investigations into lipid-immune regulation in tuberculosis.

The specific downregulation of linoleic acid in pulmonary MDMs, a core ω-6 polyunsaturated fatty acid, plays a critical role in TB immunometabolic regulation, which can be interpreted from three dimensions. First, in the arachidonic acid pathway, linoleic acid is a key precursor for prostaglandins, thromboxanes, and leukotrienes—core mediators of pulmonary inflammatory responses and Mtb intracellular survival [44]. Tbxas1 catalyzes prostaglandin H2 to thromboxane A2, which regulates macrophage phagocytosis and inflammation [45,46]. Second, M1/M2 polarization of pulmonary MDMs is closely coupled with lipid metabolism reprogramming: Acaa1b participates in lipid catabolism and PPAR signaling (a key regulator of M1/M2 switching) [47], and Acox1, a rate-limiting enzyme in peroxisomal β-oxidation, couples fatty acid metabolism with inflammation [48]. Third, competitive lipid signaling: linoleic acid metabolism competes with ω-3 polyunsaturated fatty acids for key enzymes, and Mtb-induced lipid droplet accumulation in MDMs further disrupts this balance [49]. These mechanisms suggest that linoleic acid dysregulation reshapes MDM immune function via multiple pathways.

The metabolic changes in taurocholic acid and linoleic acid in pulmonary MDMs are also regulated by systemic metabolic reprogramming induced by Mtb infection, rather than solely by intrinsic macrophage pathways. Mtb systemic infection disrupts the function of core metabolic organs such as the liver and adipose tissue [50]: taurocholic acid, mainly metabolized in the liver, may accumulate in pulmonary infection sites via the bloodstream and be taken up by MDMs, rather than being synthesized by MDMs themselves [51]. Linoleic acid levels are regulated by systemic lipid metabolism; enhanced lipolysis in adipose tissue and disrupted hepatic fatty acid metabolism reduce the systemic linoleic acid pool, decreasing MDM uptake [52]. Additionally, paracrine signals from systemically activated immune cells indirectly regulate MDM metabolite uptake, forming a complex regulatory network of local and systemic metabolism in TB.

This study has several limitations that need to be addressed. First, conclusions are based solely on transcriptomic and metabolomic correlation analyses, without validation by functional experiments such as Western blot, cytokine detection, or lipid droplet quantification. Second, the ROC results (AUC = 1.0) are exploratory, derived from a small murine cohort (n = 6 per group) using an intravenous H37Ra (attenuated strain) model, which differs from human TB (virulent strain, low-dose respiratory infection). This model induces diffuse, non-necrotic granulomas, uniform bacterial distribution, and altered macrophage activation compared to human infection [53]. Third, DEG and DAM screening relied on a single cohort without independent validation, increasing the risk of accidental results. Fourth, MDM sorting (CD45^+^F4/80^+^CD11b^+^CD11c^−^/low Siglec-F^−^Ly6C^+^) may have residual contamination by interstitial macrophages or immature monocytes, requiring single-cell RNA-seq for further verification [54].

Future studies should address these limitations to improve the clinical and scientific value of the findings. First, validate the candidate biomarkers and molecular associations using traditional aerosol infection models to mimic human natural infection [55]. Second, verify taurocholic acid and linoleic acid in human clinical samples: blood detection is feasible via HPLC-MS/MS (taurocholic acid) and GC-MS/LC-MS/MS (linoleic acid) [56], sputum detection requires optimized preprocessing to reduce interference, and BALF has high biological relevance but low accessibility due to invasive collection. Third, validate DEGs and DAMs in an independent murine cohort to enhance the result reliability. Fourth, use single-cell RNA-seq to clarify the heterogeneity of sorted MDMs [57]. Collectively, these efforts will further clarify the role of MDM linoleic acid metabolism in TB immunoregulation and promote the development of novel diagnostic strategies.

## 5. Conclusions

This study highlights Ptpn1, Dgat2, Alox5ap, Cyld, Zfp61, and Mapk11 as MDM-specific DEGs potentially involved in the immune response to tuberculosis. Metabolomic analysis identified taurocholic acid and linoleic acid as candidate biomarkers, with linoleic acid metabolism significantly linked to Tbxas1, Acaa1b, and Acox1 expression. These findings provide a foundation for exploring MDM-centered lipid metabolic pathways as potential targets for tuberculosis diagnosis and therapy.

## Figures and Tables

**Table 1:** Sequences of primers used in qRT-PCR.

Gene	Sequence of Primers
*Ptpn1*	5′-G GAACTGGGCGGCTATTTACC-3′(forward)
	5′-CAAAAGGGCTGACATCTCGGT-3′(reverse)
*Dgat2*	5′-GCGCTACTTCCGAGACTACTT-3′(forward)
	5′-GGGCCTTATGCCAGGAAACT-3′(reverse)
*Alox5ap*	5′-AGCATGAAAGCAAGGCGCATA-3′(forward)
	5′-GTACGCATCTACGCAGTTCTG-3′(reverse)
*Cyld*	5′-GGATAACCCTATTGGCAACTGG-3′(forward)
	5′-TTGGAAGTCCCTGGGATGATG-3′(reverse)
*Zfp61*	5′-ATGGTTGAGAATTTTCGGAACCT-3′(forward)
	5′-TCTGACCGTTAATCCCTGAATCT-3′(reverse)
*Mapk11*	5′-GCGGGATTCTACCGGCAAG-3′(forward)
	5′-GAGCAGACTGAGCCGTAGG-3′(reverse)
*Gapdh*	5′-AGGTCGGTGTGAACGGATTTG-3′(forward)
	5′-TGTAGACCATGTAGTTGAGGTCA-3′(reverse)

## Data Availability

All data generated or analysed during this study are included in this published article.

## Supplementary Materials

The following supporting information can be downloaded at: https://www.mdpi.com/article/10.3390/pathogens15030254/s1, Figure S1: RNA-seq saturation; Figure S2: Normality Test; Figure S3: melting curve analysis. Table S1: RNA-seq alignment rate and percentage of uniquely mapped reads; Table S2: RNA-seq ribosome alignment rate; Table S3: RNA-seq base quality; Table S4: 44 significantly correlated gene-metabolite pairs.
