# Supplementary material for: Integrating Untargeted Metabolomics and Transcriptomics in Mice with Pulmonary Tuberculosis to Reveal Changes in Linoleic Acid and Its Metabolism in Lung Monocyte-Derived Macrophages
Source: Pathogens. 2026 Feb 27;15(3):254. doi: 10.3390/pathogens15030254 (PMC13029415; doi:10.3390/pathogens15030254)
Supplement: Supplementary file 1 [file pathogens-15-00254-s001.zip › pathogens-4024977-supplementary.pdf]

Supplementary Materials

1. RNA-seq Data Quality Analysis

① RNA-seq alignment rate and percentage of uniquely mapped reads

Table S1. RNA-seq alignment rate and percentage of uniquely mapped reads.

Sample	Total	Unmapped (%)	Unique_Mapped (%)	Multiple_Mapped (%)	Total_Mapped (%)
C1	44143846	2728021 (6.18%)	38273685 (86.70%)	3142140 (7.12%)	41415825 (93.82%)
C2	50876060	2856741 (5.62%)	44625681 (87.71%)	3393638 (6.67%)	48019319 (94.38%)
C3	46117104	2611384 (5.66%)	40348395 (87.49%)	3157325 (6.85%)	43505720 (94.34%)
T1	58328694	6336059 (10.86%)	48273982 (82.76%)	3718653 (6.38%)	51992635 (89.14%)
T2	42087692	4012614 (9.53%)	35843699 (85.16%)	2231379 (5.30%)	38075078 (90.47%)
T3	45619752	5747884 (12.60%)	37415743 (82.02%)	2456125 (5.38%)	39871868 (87.40%)

② RNA-seq ribosome alignment rate

Table S2. RNA-seq ribosome alignment rate.

Sample	clean_reads	Mapped_Reads(%)	Unmapped_Reads(%)
C1	45315356	1171510 (2.59%)	44143846 (97.41%)
C2	52019246	1143186 (2.20%)	50876060 (97.80%)
C3	47163516	1046412 (2.22%)	46117104 (97.78%)
T1	62186630	3857936 (6.20%)	58328694 (93.80%)
T2	44499746	2412054 (5.42%)	42087692 (94.58%)
T3	48363706	2743954 (5.67%)	45619752 (94.33%)

③ RNA-seq base quality

Table S3. RNA-seq base quality.

Sample	RawData(bp)	BF_Q20(%)	BF_Q30(%)	BF_N(%)	BF_GC(%)	CleanData(bp)	AF_Q20(%)	AF_Q30(%)	AF_N (%)	AF_GC(%)
C1	6955375200	6696914371 (96.28%)	6397652438 (91.98%)	93659 (0.00%)	3470363682 (49.89%)	6204687882	6032610548 (97.23%)	5780659346 (93.17%)	48282 (0.00%)	3071973038 (49.51%)
C2	7922354100	7711543409 (97.34%)	7422283409 (93.69%)	164463 (0.00%)	3967974244 (50.09%)	7430604645	7271068518 (97.85%)	7012999976 (94.38%)	51198 (0.00%)	3705722393 (49.87%)
C3	7175216400	6982850091 (97.32%)	6707534105 (93.48%)	93524 (0.00%)	3587446996 (50.00%)	6749420322	6593564560 (97.69%)	6346146918 (94.03%)	38610 (0.00%)	3360406200 (49.79%)
T1	9886874400	9398118135 (95.06%)	8933814981 (90.36%)	78525 (0.00%)	5004673237 (50.62%)	7794729827	7473330293 (95.88%)	7145707481 (91.67%)	59451 (0.00%)	3877444846 (49.74%)
T2	6952812000	6643654169 (95.55%)	6318158067 (90.87%)	54729 (0.00%)	3604104635 (51.84%)	5894137258	5674555892 (96.27%)	5420385075 (91.96%)	43720 (0.00%)	3042091581 (51.61%)
T3	7591995600	7211635736 (94.99%)	6861169004 (90.37%)	61076 (0.00%)	3846719542 (50.67%)	6275348765	6034930072 (96.17%)	5774554156 (92.02%)	47738 (0.00%)	3150082018 (50.20%)

④ RNA-seq saturation

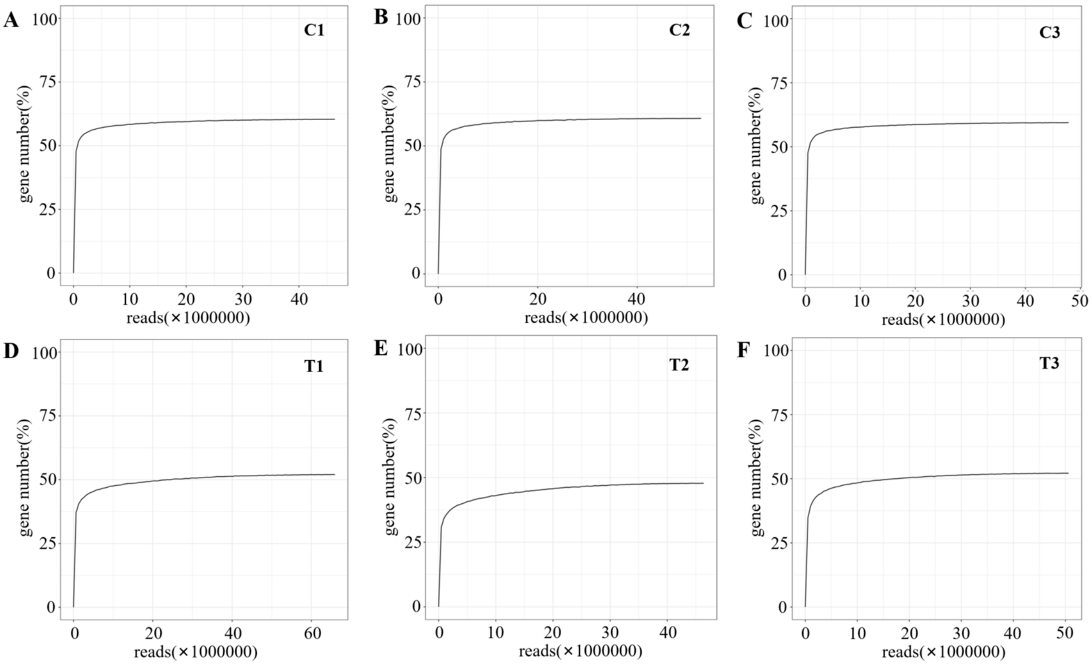

Figure S1. RNA-seq saturation. (A-F) show the sequencing saturation of C1, C2, C3, T1, T2 and T3, respectively, where C denotes the control group and T denotes the H37Ra-infected group.

Table S4. 44 significantly correlated gene-metabolite pairs.

Gene id	Gene	Metabolite	r	95%CI	p value	FDR value
ENSMUSG00000022793	B4galt4	Linoleic acid	0.9297	0.4814 to 0.9925	0.0072	0.0282
ENSMUSG00000021665	Hexb	Linoleic acid	0.9169	0.4115 to 0.9910	0.0101	0.0282
ENSMUSG00000074207	Adh1	Linoleic acid	0.8586	0.1553 to 0.9843	0.0286	0.0393
ENSMUSG00000035778	Ggta1	Linoleic acid	0.9532	0.6253 to 0.9950	0.0032	0.0249
ENSMUSG00000017969	Ptgis	Linoleic acid	0.8955	0.3073 to 0.9886	0.0158	0.0331
ENSMUSG00000061740	Cyp2d22	Linoleic acid	0.9584	0.6609 to 0.9956	0.0026	0.0249
ENSMUSG00000029925	Tbxas1	Linoleic acid	0.8835	0.2544 to 0.9872	0.0196	0.0348
ENSMUSG00000026003	Acadl	Linoleic acid	0.9419	0.5533 to 0.9938	0.005	0.0249
ENSMUSG00000025150	Cbr2	Linoleic acid	0.8244	0.03877 to 0.9802	0.0435	0.0466

ENSMUSG00000032487	Ptgs2	Linoleic acid	0.8857	0.2635 to 0.9875	0.0189	0.0348
ENSMUSG00000003809	Gcdh	Linoleic acid	0.954	0.6312 to 0.9951	0.0031	0.0249
ENSMUSG00000032323	Cyp11a1	Linoleic acid	0.8827	0.2508 to 0.9871	0.0198	0.0348
ENSMUSG00000032047	Acat1	Linoleic acid	0.8226	0.03332 to 0.9800	0.0444	0.0466
ENSMUSG00000006731	B4galnt1	Linoleic acid	-0.8619	-0.9847 to -0.1676	0.0273	0.0393
ENSMUSG00000024900	Cpt1a	Linoleic acid	0.9412	0.5491 to 0.9937	0.0051	0.0249
ENSMUSG00000033105	Lss	Linoleic acid	0.9446	0.5699 to 0.9941	0.0045	0.0249
ENSMUSG00000021759	Plpp1	Linoleic acid	0.8372	0.07994 to 0.9817	0.0376	0.0466
ENSMUSG00000035596	Mboat7	Linoleic acid	-0.848	-0.9830 to -0.1168	0.0329	0.0439
ENSMUSG00000028603	Scp2	Linoleic acid	0.8224	0.03248 to 0.9799	0.0445	0.0466
ENSMUSG00000020777	Acox1	Linoleic acid	0.9468	0.5836 to 0.9943	0.0042	0.0249
ENSMUSG00000000594	Gm2a	Linoleic acid	0.8307	0.05871 to 0.9809	0.0406	0.0466
ENSMUSG00000028145	Them4	Linoleic acid	0.9547	0.6354 to 0.9952	0.003	0.0249
ENSMUSG00000027195	Hsd17b12	Linoleic acid	0.8914	0.2888 to 0.9881	0.017	0.034
ENSMUSG00000026922	Agpat2	Linoleic acid	-0.871	-0.9858 to -0.2028	0.0239	0.038
ENSMUSG00000020828	Pld2	Linoleic acid	0.9166	0.4101 to 0.9910	0.0101	0.0282
ENSMUSG00000028670	Lypla2	Linoleic acid	-0.87	-0.9856 to -0.1990	0.0242	0.038
ENSMUSG00000038007	Acer2	Linoleic acid	0.8673	0.1883 to 0.9853	0.0252	0.0382
ENSMUSG00000037366	Pafah2	Linoleic acid	0.9135	0.3943 to 0.9906	0.0109	0.0282
ENSMUSG00000003363	Pld3	Linoleic acid	-0.8998	-0.9891 to -0.3271	0.0146	0.0331
ENSMUSG00000028541	B4galt2	Linoleic acid	0.8174	0.01724 to 0.9793	0.047	0.0481
ENSMUSG00000055301	Adh7	Linoleic acid	0.9214	0.4357 to 0.9915	0.009	0.0282
ENSMUSG00000063275	Hacd1	Linoleic acid	0.9272	0.4673 to 0.9922	0.0078	0.0282
ENSMUSG00000020745	Pafah1b1	Linoleic acid	0.9939	0.9429 to 0.9994	＜0.0001	＜0.0001
ENSMUSG00000021036	Sptlc2	Linoleic acid	0.8776	0.2295 to 0.9865	0.0216	0.0366

ENSMUSG00000017715	Pgs1	Linoleic acid	-0.9211	-0.9915 to -0.4337	0.0091	0.0282
ENSMUSG00000030760	Acer3	Linoleic acid	0.8344	0.07074 to 0.9814	0.0389	0.0466
ENSMUSG00000071072	Ptges3	Linoleic acid	0.897	0.3140 to 0.9888	0.0154	0.0331
ENSMUSG00000010651	Acaa1b	Linoleic acid	0.9138	0.3956 to 0.9907	0.0108	0.0282
ENSMUSG00000031903	Pla2g15	Linoleic acid	0.8228	0.03386 to 0.9800	0.0443	0.0466
ENSMUSG00000006390	Elovl1	Linoleic acid	0.8268	0.04647 to 0.9805	0.0424	0.0466
ENSMUSG00000024781	Lipa	Linoleic acid	0.8272	0.04754 to 0.9805	0.0422	0.0466
ENSMUSG00000042289	Hsd3b7	Linoleic acid	0.8961	0.3101 to 0.9887	0.0156	0.0331
ENSMUSG00000031467	Agpat5	Linoleic acid	0.8118	0.00072 to 0.9786	0.0498	0.0498
ENSMUSG00000028138	Adh5	Linoleic acid	0.8598	0.1594 to 0.9844	0.0281	0.0393

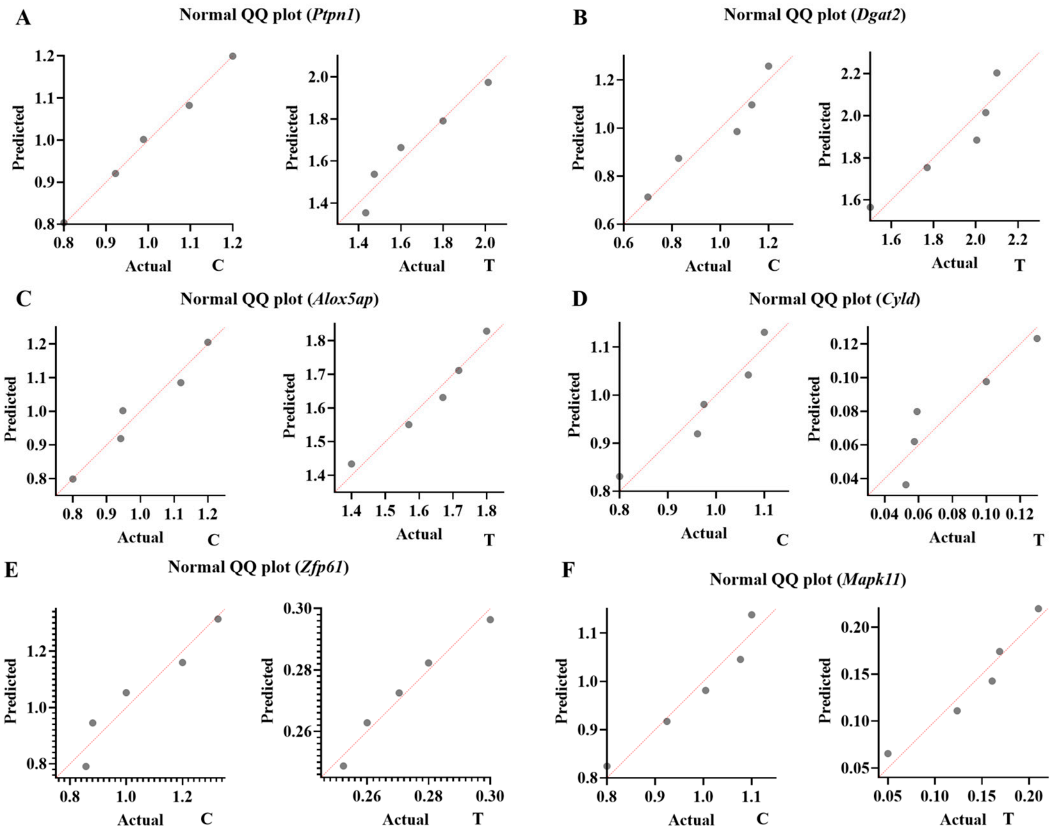

Figure S2. Normality Test. Normality of the quantitative data in Figures 5B－G was assessed by the Shapiro-Wilk test. All datasets followed a normal distribution (P>0.05), meeting the prerequisite assumption for the t-test.

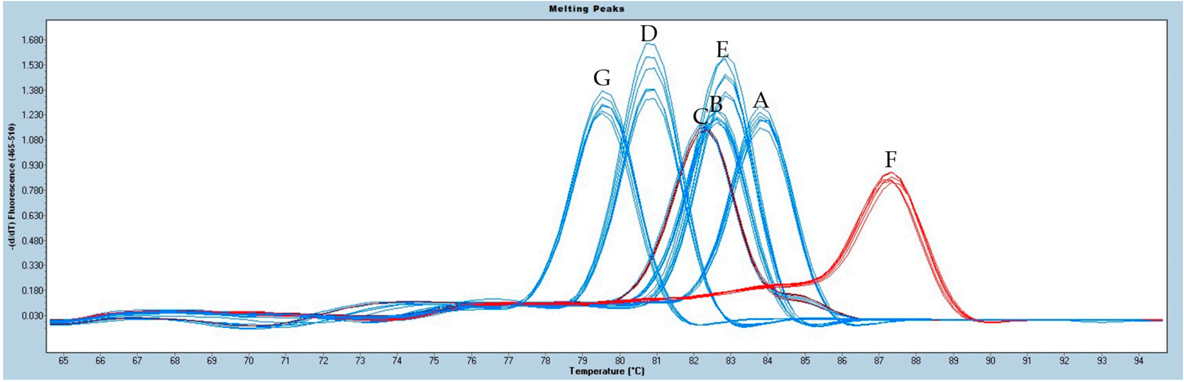

Figure S3. melting curve analysis. Panels A－G in the figure represent the melting curves of the primers for *Gapdh*, *Alox5ap*, *Ptpn1*, *Zfp61*, *Dagat2*, *Mapk11*, and *Cyld*, respectively. Melting curve analysis was performed, which revealed that all target genes exhibited a single sharp melting peak with no non-specific peaks or primer-dimer signals detected.
